# Supplementary material for: Genetic evidence supports the development of SLC26A9 targeting therapies for the treatment of lung disease
Source: NPJ Genom Med. 2022 Apr 8;7:28. doi: 10.1038/s41525-022-00299-9 (PMC8993824; doi:10.1038/s41525-022-00299-9)
Supplement: Supplementary file 1 — Supplementary Materials for “Genetic evidence supports the development of SLC26A9 targeting therapies for the treatment of lung disease” [file 41525_2022_299_MOESM1_ESM.docx]

**Supplementary Materials for**

“**­­Genetic evidence supports the development of SLC26A9 targeting therapies for the treatment of lung disease­”**

Jiafen Gong^1^, Gengming He^1,2^, Cheng Wang^1^, Claire Bartlett^3^, Naim Panjwani^1^, Scott Mastromatteo^1^, Fan Lin^1^, Katherine Keenan^1,3^, Julie Avolio^3^, Anat Halevy^1^, Michelle Shaw^3^, Mohsen Esmaeili^1^, Guillaume Cote Maurais^4^, Damien Adam^4,5^, Stéphanie Bégin^4^, Candice Bjornson^6^, Mark Chilvers^7^, Joe Reisman^8^, April Price^9^, Michael Parkins^10^, Richard van Wylick^11^, Yves Berthiaume ^5^, Lara Bilodeau^12^, Dimas Mateos-Corral^13^, Daniel Hughes^13^, Mary J. Smith^14^, Nancy Morrison^15^, Janna Brusky^16^, Elizabeth Tullis^17^, Anne L. Stephenson^17^, Bradley S. Quon^18^, Pearce Wilcox^18^, Winnie M. Leung^19^, Melinda Solomon^20,21^, Lei Sun ^2,22^, Emmanuelle Brochiero^4,5^, Theo J. Moraes^3,20^, Tanja Gonska^3,23^, Felix Ratjen^3,21^, Johanna M. Rommens^1,24^, Lisa J. Strug^1,2,22,25, 26,*^

^1^Program in Genetics and Genome Biology, The Hospital for Sick Children; Toronto, ON, Canada.

^2^Biostatistics Division, Dalla Lana School of Public Health, University of Toronto; Toronto, ON, Canada.

^3^Translational Medicine, The Hospital for Sick Children; Toronto, ON, Canada.

^4^CRCHUM; Montréal, QC, Canada.

^5^Department of Medicine, Faculty of Medicine, Université de Montréal; Montréal, QC, Canada.

^6^Alberta Children’s Hospital; Calgary, AB, Canada.

^7^ British Columbia Children’s Hospital; Vancouver, BC, Canada.

^8^The Children’s Hospital of Eastern Ontario; Ottawa, ON, Canada.

^9^ The Children’s Hospital, London Health Science Centre; London, ON, Canada.

^10^ Foothills Medical Centre; Calgary, AB, Canada.

^11^ Kingston Health Sciences Centre; Kingston, ON, Canada.

^12^Centre de recherche de l’Institut universitaire de cardiologie et de pneumologie de Québec-Université Laval; Québec City, QC, Canada.

^13^IWK Health Centre; Halifax, NS, Canada.

^14^ Faculty of Medicine, Memorial University of Newfoundland; St. John’s, NL, Canada.

^15^Queen Elizabeth II Health Sciences Centre; Halifax, NS, Canada.

^16^Department of Pediatrics, University of Saskatchewan; Saskatoon, SK, Canada.

^17^St. Michael’s Hospital; Toronto, ON, Canada.

^18^St. Paul’s Hospital; Vancouver, BC, Canada.

^19^ University of Alberta Hospital; Edmonton, AB, Canada.

^20^Respiratory Medicine, Hospital for Sick Children; Toronto, ON, Canada.

^21^Department of Paediatrics, University of Toronto; Toronto, ON, Canada.

^22^Department of Statistical Sciences, University of Toronto; Toronto, ON, Canada.

^23^Division of Gastroenterology, Hepatology and Nutrition, The Hospital for Sick Children; Toronto, ON, Canada.

^24^Department of Molecular Genetics, University of Toronto; Toronto, ON, Canada.

^25^The Centre for Applied Genomics, Hospital for Sick Children; Toronto, ON, Canada.

^26^Department of Computer Science, University of Toronto; Toronto, ON, Canada.

^*^Corresponding author: Lisa J. Strug, PhD

Program in Genetics and Genome Biology

SickKids Research Institute

Room 12.9705, PGCRL

686 Bay Street

Toronto, ON

M5G 0A4

Email: [lisa.strug@utoronto.ca](mailto:lisa.strug@utoronto.ca)

Tel: +1416-813-7654 X301762

# **Supplementary Note**

**Haplotype association with Saknorm in CGMS participants homozygous for Phe508del**

Constructing haplotypes in the CGMS in individuals homozygous for Phe508del as in Saknorm variation (Supplementary Table 3), although restricting to the subset in the CGMS of individuals with either LR/LR, LR/HR or HR/HR (n=286) does demonstrate significant association evidence (effect size=0.14; p=0.026). Since in this analysis the LR and HR haplotypes are completely tagged by the C and T alleles of rs7512462, the haplotype analysis in this subset of n=286 is equivalent to the association of Saknorm with rs7512462 genotype.

Comparing the significant SNP (rs7512462) analysis with the results of the haplotype analysis, we note that although the LR and HR haplotypes are completely tagged, respectively, by the C and T alleles, the reverse is not true. Specifically, the C allele appears on another haplotype, while the T allele appears on five other haplotypes reported in (1). As a result, the HR haplotype analysis uses a comparison haplotype group that includes some haplotypes with the rs7512462 T risk allele and this appears to attenuate the results. The haplotype analysis of this locus akin to that conducted in (1) does not provide greater power than using the single SNP rs7512462 to mark the locus, so all primary analyses focus on the rs7512462 genotype.

# **Supplementary References**

1. Lam AN, Aksit MA, Vecchio-Pagan B, Shelton CA, Osorio DL, Anzmann AF, et al. Increased expression of anion transporter SLC26A9 delays diabetes onset in cystic fibrosis. J Clin Invest. 2020;130(1):272-86.

2. Taylor C, Commander CW, Collaco JM, Strug LJ, Li W, Wright FA, et al. A novel lung disease phenotype adjusted for mortality attrition for cystic fibrosis Genetic modifier studies. Pediatr Pulmonol. 2011;46(9):857-69.

3. Corvol H, Mesinele J, Douksieh IH, Strug LJ, Boelle PY, Guillot L. SLC26A9 Gene Is Associated With Lung Function Response to Ivacaftor in Patients With Cystic Fibrosis. Front Pharmacol. 2018;9:828.

4. Eastman AC, Pace RG, Dang H, Aksit MA, Vecchio-Pagan B, Lam AN, et al. SLC26A9 SNP rs7512462 is not associated with lung disease severity or lung function response to ivacaftor in cystic fibrosis patients with G551D-CFTR. J Cyst Fibros. 2021.

5. Strug LJ, Gonska T, He G, Keenan K, Ip W, Boelle PY, et al. Cystic fibrosis gene modifier SLC26A9 modulates airway response to CFTR-directed therapeutics. Hum Mol Genet. 2016;25(20):4590-600.

6. Gong J, Wang F, Xiao B, Panjwani N, Lin F, Keenan K, et al. Genetic association and transcriptome integration identify contributing genes and tissues at cystic fibrosis modifier loci. PLoS Genet. 2019;15(2):e1008007.

# **Supplementary Figures**


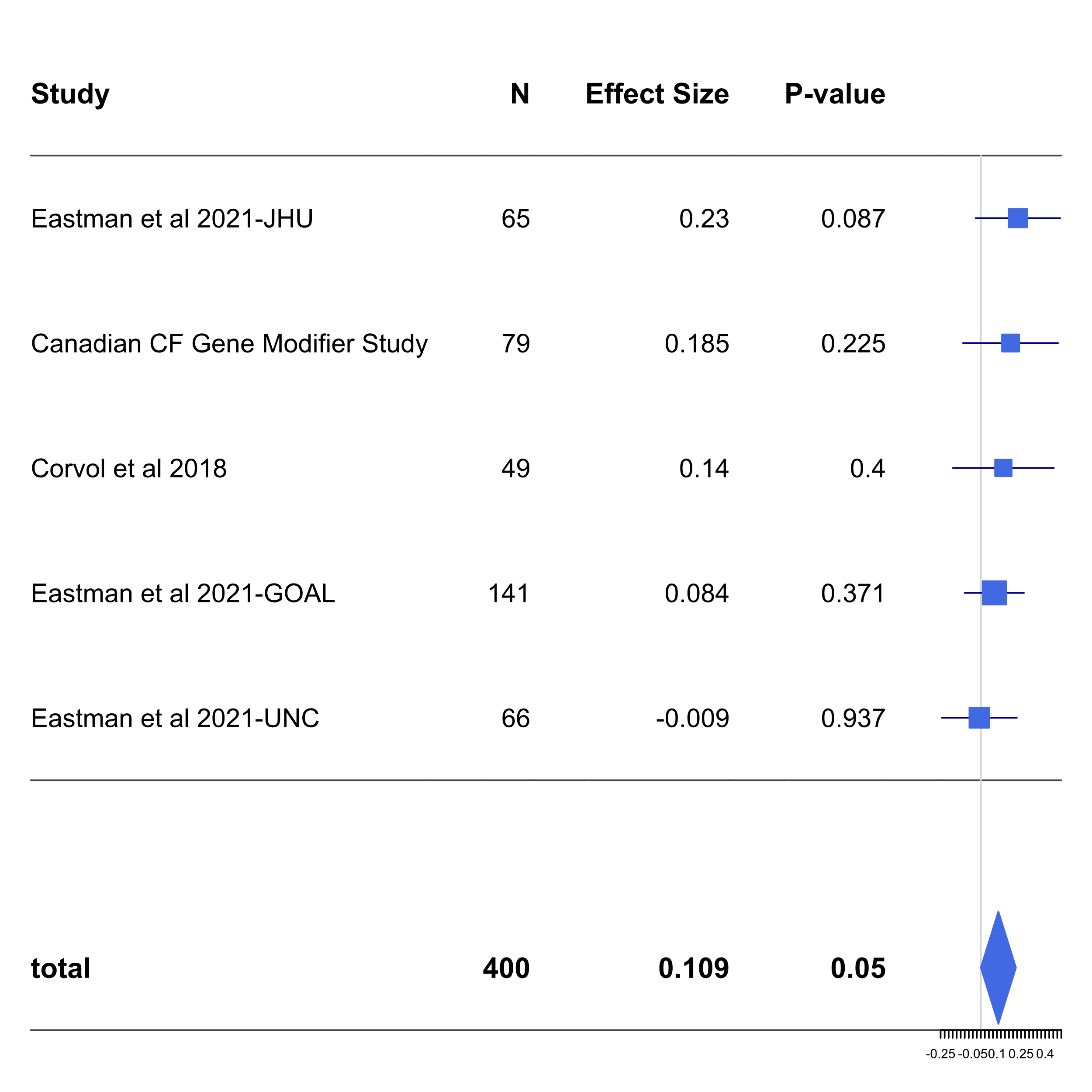


**Supplementary Figure 1.** Forest plot of association between rs7512462 and lung function, measured as Saknorm (2) in samples with at least one G551D variant. Saknorm is calculated using FEV_1_ measured prior to modulator treatment, if applicable. The Canadian CF Gene Modifier Study (CGMS) association is meta-combined with results from other published studies (Corvol et al 2018 (3) and Eastman et al 2021 (4), where Eastman et al 2021 includes three sub-studies, GOAL, UNC and JHU). The CGMS sample here includes 54 individuals included previously in (5) and 25 individuals newly recruited into the CGMS and not previously included in a publication. The inverse variance weighted meta-analysis is reported here.


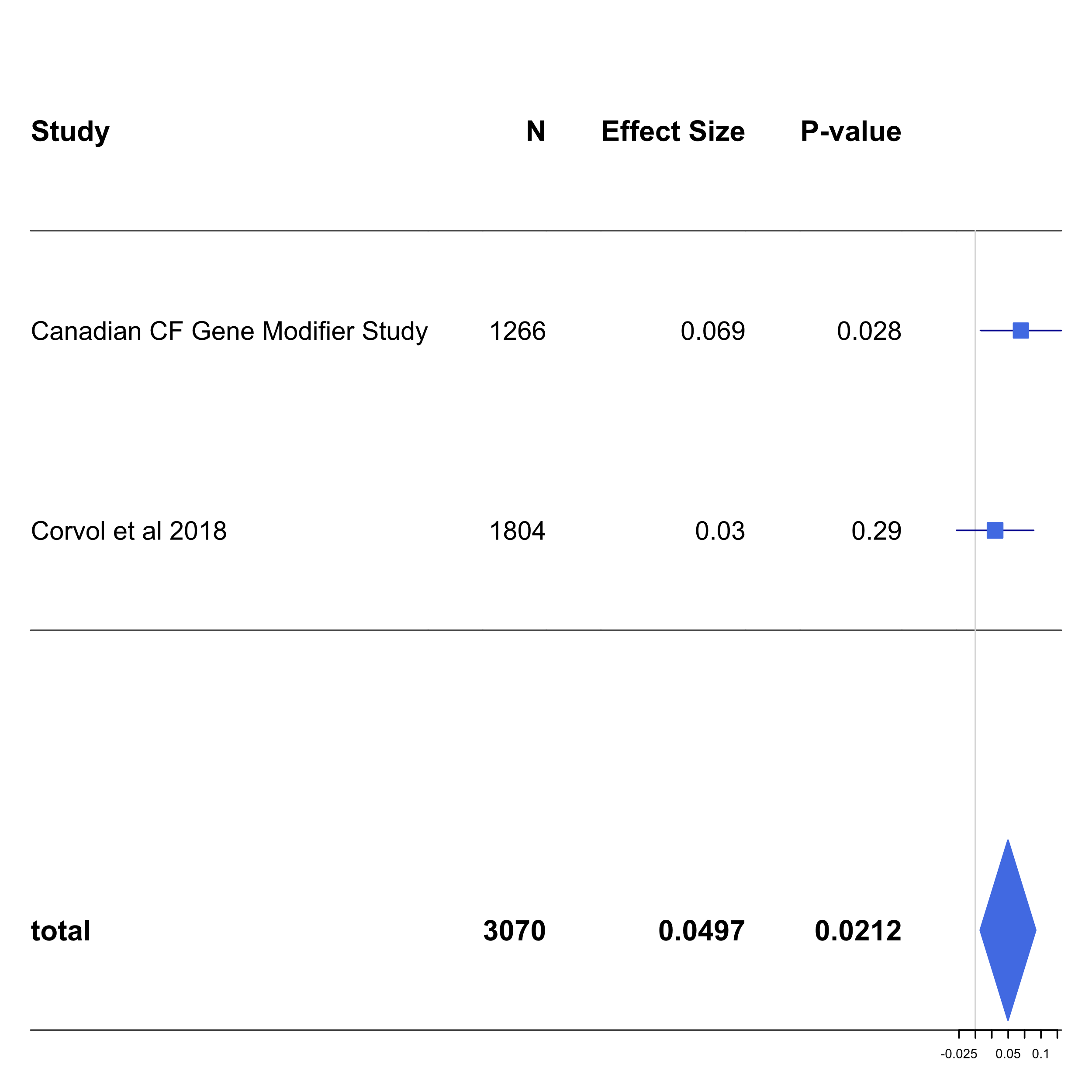


**Supplementary Figure 2**. Forest plot of association between rs7512462 and lung function, measured as Saknorm (2) in individuals homozygous for Phe508del. Saknorm is calculated using FEV_1_ measurements prior to modulator treatment, if applicable. The Canadian CF Gene Modifier Study (CGMS) association is meta-combined with results from the French Gene Modifier Study (Corvol et al 2018; (3)). The CGMS sample here includes 1,013 individuals previously reported in (5) and 253 individuals newly recruited into the CGMS and not previously included in a publication. The result from inverse variance weighted meta-analysis is reported here.


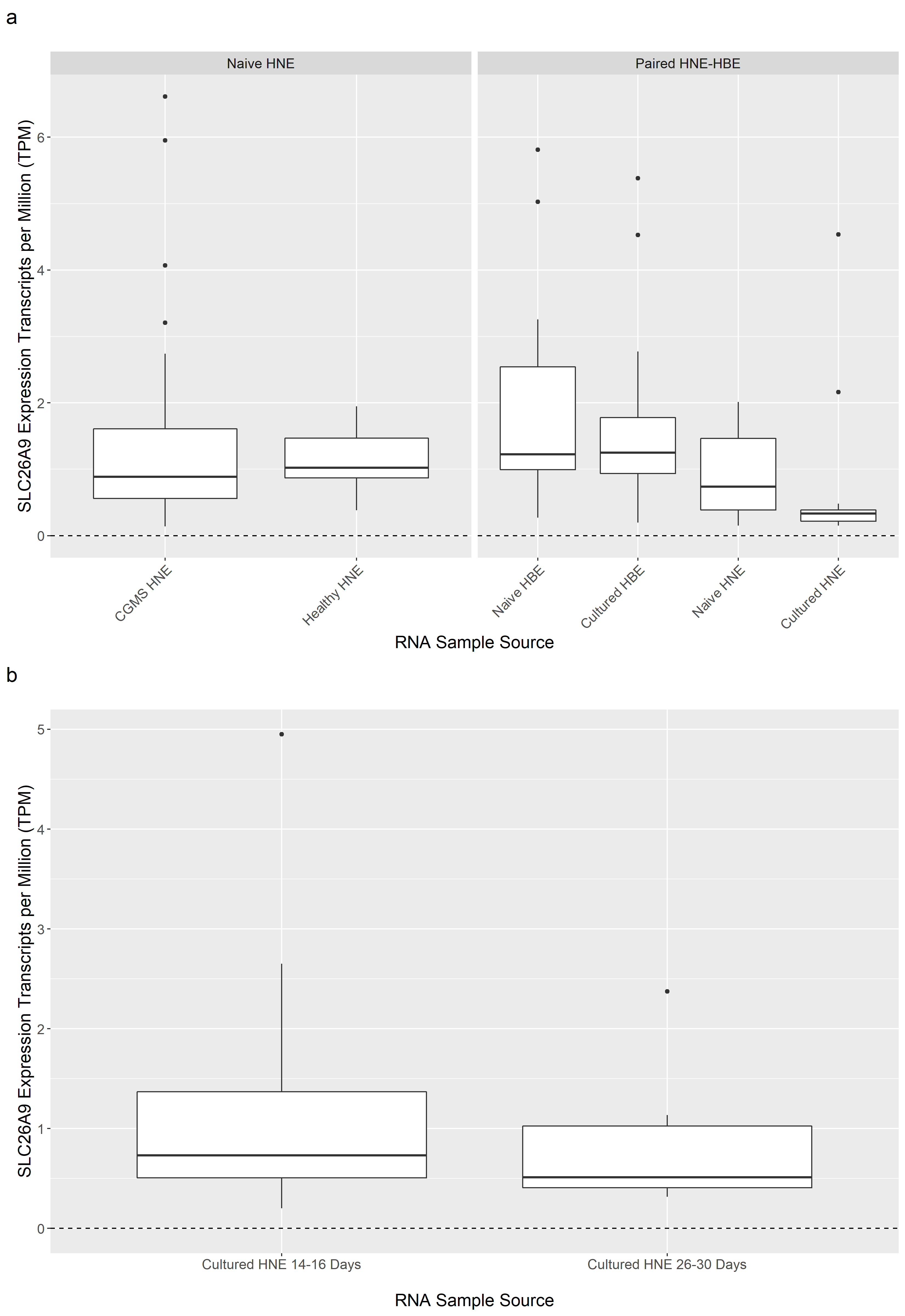


**Supplementary Figure 3.** *SLC26A9* gene expression in various tissue models to guide functional studies. (a) *SLC26A9* expression is low in the bulk RNA-Seq of CGMS CF participants and healthy controls in naïve HNE (left), with diminishing expression with culturing of both HNE and HBE (right). (b) *SLC26A9* gene expression in cultured naïve nasal cells from the same individuals (n=9) in CGMS at two different time points. Both sets of cultures were P3.

# **Supplementary Tables**

**Supplementary Table 1:** Number of participants removed according to the exclusion criteria for the CFTR modulator study. Sample exclusion is in the order of CFTR mutation eligibility for drug, FEV_1pp_ baseline measurement, post-treatment FEV_1pp_ measurement, non-commercial modulator dosage and genotype quality control as shown in the last four columns in the table. All participants included had CFTR genotype approved for the corresponding modulator (at least one gating variant for IVA and homozygous Phe508del for LUM/IVA), and were on the commercial dose, although some of the CGMS participants may have originally gained access to the CFTR modulators through participation in the clinical trial or through compassionate use.

| Studies | CFTR genotype not eligible for drug | FEV_1pp_ baseline missing or measured >3 month of treatment initiation | FEV_1pp_ baseline outside [30,96] | Missing post-treatment FEV_1pp_ measure | Non-commercial modulator dosage | Not genotyped | Genotyping missing rate >10% | Sex mismatch | Non- European ancestry |
| --- | --- | --- | --- | --- | --- | --- | --- | --- | --- |
| PROSPECT | 0 | 0 | 58 | 9 | 0 | 0 | 1 | 2 | 7 |
| IVA in CGMS | 15 | 15 | 17 | 1 | 0 | 2 | 0 | 0 | 0 |
| LUM/IVA in CGMS | 2 | 48 | 43 | 67 | 5 | 5 | 0 | 1 | 0 |

**Supplementary Table 2**: Meta-analysis for the association between rs7512462 and lung function prior to treatment with Ivacaftor. Lung function is measured as Saknorm which is a continuous FEV_1_- based CF-specific percentile that is normalized and accounts for age, sex and height, and is adjusted for survival time (2). For individuals with at least one G551D mutations, the Canadian CF Gene Modifier Study (CGMS) association is meta-combined with results from Corvol et al 2018 (3) and Eastman et al 2021 (4) which includes three sub-studies: GOAL, UNC and JHU. For samples with homozygous Phe508del or at least one copy of Gating mutation approved for IVA, the Canadian CF Gene Modifier Study (CGMS) association is meta-combined with results from Corvol et al 2018 (3). Results from inverse variance weighted and sample size weighted meta-analysis are reported.

|  |  |  |  |  | Inverse-variance weighted  meta-analysis | | Sample size weighted  meta-analysis | |
| --- | --- | --- | --- | --- | --- | --- | --- | --- |
| CFTR group | Effect size | P-value | N | Study | Effect size | P-value | Effect size | P-value |
| G551D/other | 0.230 | 0.087 | 65 | Eastman et al 2021-JHU | 0.109 | 0.050 | 0.119 | 0.036 |
|  | 0.185 | 0.225 | 79 | CGMS |  |  |  |  |
|  | 0.140 | 0.400 | 49 | Corvol et al 2018 |  |  |  |  |
|  | 0.084 | 0.371 | 141 | Eastman et al 2021-GOAL |  |  |  |  |
|  | -0.009 | 0.937 | 66 | Eastman et al 2021-UNC |  |  |  |  |
| Phe508del/Phe50del | 0.030 | 0.290 | 1804 | Corvol et al 2018 | 0.050 | 0.021 | 0.046 | 0.037 |
|  | 0.069 | 0.028 | 1266 | CGMS |  |  |  |  |
| Gating/other | 0.130 | 0.270 | 93 | Corvol et al 2018 | 0.123 | 0.176 | 0.122 | 0.183 |
|  | 0.114 | 0.417 | 89 | CGMS |  |  |  |  |

**Supplementary Table 3**. Haplotype association with Saknorm in individuals in the CGMS who are homozygous Phe508del. The haplotypes are constructed in the region chr1: 205899595-205921859 (hg19) as defined in (1) from imputed genotypes (6). The 8 haplotypes defined in (1) are analyzed here, excluding one multi-allelic variant rs144469431. The first row corresponds to the low risk (LR) haplotype defined in (1) while the last row represents the high risk (HR) haplotype defined in (1). The association results from two analyses are presented: the first is from an analysis that uses the same PLINK command as implemented in (1) (PLINK v1.07 with options --chap and --each-vs-others) in 1,164 unrelated CGMS participants with Phe508del/Phe508del and the second is from a linear regression with a robust variance estimator to account for the inclusion of related individuals in 1,266 CGMS participants with Phe508del/Phe508del.

|  | Haplotype association CGMS Unrelated Phe508del/Phe508del (n=1164) | | | Haplotype association CGMS Related Phe508del/Phe508del (n=1266) | | |
| --- | --- | --- | --- | --- | --- | --- |
| Forty SNP haplotype (chr1: 205899595-205921859) | MHF | Effect size | P-value | MHF | Effect Size | P-value |
| GGCAGCGCGCAAGTGCAATAAGTTCCATATTCCAAGCCCC | 0.267 | (-ref-) | 0.333 | 0.251 | NA | 0.205 |
| GGCAGCGCGCAAGTGCAATAAGCTCCAACGCCCGGGCCCT | 0.047 | 0.107 | 0.121 | 0.042 | 0.152 | 0.027 |
| AGAA-CGGTCAAGTACAATAGACATTGACGTCTGGGCCCC | 0.025 | 0.010 | 0.764 | 0.022 | -0.102 | 0.339 |
| AGAA-CGGTCAAGTACAATAGACATTGACGTCTGGGCCCT | 0.081 | -0.033 | 0.719 | 0.081 | -0.037 | 0.612 |
| AGAAGCGGGCAGT-ACACTAGACATTGACGCCCGGCCCCC | 0.016 | -0.042 | 0.745 | 0.036 | -0.094 | 0.317 |
| AGAA-GGGGCAGT-ATGCAAGACTTTGACGCTCGGGCCCC | 0.073 | -0.025 | 0.791 | 0.064 | 0.014 | 0.740 |
| ACCT-GAGTGGGT-ATGCAAGACATTGACGCCCGGCGTTT | 0.038 | -0.041 | 0.714 | 0.025 | -0.019 | 0.989 |
| ACCT-GAGTGGGT-ATGCACGACATTGACGCTCGGCGTTT | 0.218 | -0.026 | 0.645 | 0.212 | -0.039 | 0.309 |

**Supplementary Table 4.** Association analysis using a linear mixed-effect model with a random intercept with n=45 individuals on ivacaftor with multiple follow-up measures within [15,400] days. Rs7512462 is coded recessively and covariates include FEV_1pp_ and age at baseline, an indicator for whether the participant was in the previous published study ((5); Early CGMS cohort), the number of days between baseline measurement and treatment initiation (Days from baseline measure to treatment), and number of days between treatment initiation and each FEV_1pp_ measurement on treatment (Days from treatment to each treatment measure).

|  | Effect size | S. E | t value | P-value |
| --- | --- | --- | --- | --- |
| rs7512462_CC | 12.759 | 5.471 | 2.332 | 0.025 |
| Fev1pp at baseline | -0.005 | 0.082 | -0.058 | 0.954 |
| Age at baseline | -0.062 | 0.109 | -0.574 | 0.569 |
| Early CGMS cohort | 2.951 | 2.896 | -1.019 | 0.315 |
| Days from baseline measure to treatment initiation | -0.132 | 0.066 | -2.002 | 0.052 |
| Days from treatment to each treatment measure | 0.003 | 0.005 | 0.665 | 0.507 |
