# Supplementary material for: Genetic evidence supports the development of SLC26A9 targeting therapies for the treatment of lung disease
Source: NPJ Genom Med. 2022 Apr 8;7:28. doi: 10.1038/s41525-022-00299-9 (PMC8993824; doi:10.1038/s41525-022-00299-9)
Supplement: Supplementary file 2 — Reporting Summary [file 41525_2022_299_MOESM2_ESM.pdf]

nature portfolio

Corresponding author(s): Lisa Strug

Last updated by author(s): Dec 30,2021

Reporting Summary

Nature Portfolio wishes to improve the reproducibility of the work that we publish. This form provides structure for consistency and transparency in reporting. For further information on Nature Portfolio policies, see our Editorial Policies and the Editorial Policy Checklist.

Statistics

For all statistical analyses, confirm that the following items are present in the figure legend, table legend, main text, or Methods section.

n/a	Confirmed	
☐	☒	The exact sample size (*n*) for each experimental group/condition, given as a discrete number and unit of measurement
☐	☒	A statement on whether measurements were taken from distinct samples or whether the same sample was measured repeatedly
☐	☒	The statistical test(s) used AND whether they are one- or two-sided<br>*Only common tests should be described solely by name; describe more complex techniques in the Methods section.*
☐	☒	A description of all covariates tested
☐	☒	A description of any assumptions or corrections, such as tests of normality and adjustment for multiple comparisons
☐	☒	A full description of the statistical parameters including central tendency (e.g. means) or other basic estimates (e.g. regression coefficient) AND variation (e.g. standard deviation) or associated estimates of uncertainty (e.g. confidence intervals)
☐	☒	For null hypothesis testing, the test statistic (e.g. *F*, *t*, *r*) with confidence intervals, effect sizes, degrees of freedom and *P* value noted<br>*Give P values as exact values whenever suitable.*
☒	☐	For Bayesian analysis, information on the choice of priors and Markov chain Monte Carlo settings
☒	☐	For hierarchical and complex designs, identification of the appropriate level for tests and full reporting of outcomes
☐	☒	Estimates of effect sizes (e.g. Cohen's *d*, Pearson's *r*), indicating how they were calculated

Our web collection on statistics for biologists contains articles on many of the points above.

Software and code

Policy information about availability of computer code

Data collection	no software was used
Data analysis	All code and analyses steps implemented to process the UK Biobank data are available at https://github.com/strug-hub/UKBB_spirometry_on_COPD. The software used for colocalization plotting and statistical testing is available at https://locusfocus.research.sickkids.ca/. The other analyses did not require any custom codes and are described in the Methods Section.

For manuscripts utilizing custom algorithms or software that are central to the research but not yet described in published literature, software must be made available to editors and reviewers. We strongly encourage code deposition in a community repository (e.g. GitHub). See the Nature Portfolio guidelines for submitting code & software for further information.

Data

Policy information about availability of data

All manuscripts must include a data availability statement. This statement should provide the following information, where applicable:

- Accession codes, unique identifiers, or web links for publicly available datasets
- A description of any restrictions on data availability
- For clinical datasets or third party data, please ensure that the statement adheres to our policy

The datasets generated and/or analyzed in this paper are publicly available. Data from the CGMS analyzed for the lung function pre- and post-modulator treatment are available from the Canadian CF registry at https://www.cysticfibrosis.ca/our-programs/cf-registry/requesting-canadian-cf-registry-data; the functional data and RNA-seq data from CGMS is available from the CFIT program at https://lab.research.sickkids.ca/cfit/cystic-fibrosis-patients-families-researchers/, and the paired cultured and fresh naïve HNE and HBE is available at GEO (GSE172232). The US PROSPECT data are available by application to the US CFF at https://www.cff.org/researchers/cf-foundation-biorepository and the study is registered on https://clinicaltrials.gov/ct2/show/NCT02477319. The single-cell RNA-sequencing data are

downloaded from the Human Lung Cell Atlas at http://hlca.ds.czbiohug.org. The summary statistics for the pheWAS study is available at https://atlas.ctglab.nl/PheWAS and the meconium ileus association results that have been used for colocalization analysis can be downloaded at https://lab.research.sickkids.ca/strug/publications-software/. The data used for the COPD analysis are available through application to the UK Biobank.

Field-specific reporting

Please select the one below that is the best fit for your research. If you are not sure, read the appropriate sections before making your selection.

☒ Life sciences ☐ Behavioural & social sciences ☐ Ecological, evolutionary & environmental sciences

For a reference copy of the document with all sections, see nature.com/documents/nr-reporting-summary-flat.pdf

Life sciences study design

All studies must disclose on these points even when the disclosure is negative.

Sample size	Sample size calculations were not carried out ahead of time; in all analyses we used all samples available to us through the Canadian CF Gene Modifier Study, the US PROSPECT Study or the other resources we used.
Data exclusions	All the data exclusion criteria used in this paper align with previous publications by us and others. All samples included into the analyses passed genotype QC as mentioned in Gong et al 2019, ie. samples with high missing rate , sex mismatch between genotype and reported clinical records, samples clustered with non-European samples were excluded. Lung function severity in the absence of modulator treatment for participants in CGMS was measured by Saknorm, which is survival adjusted average CF-specific Kulich FEV1 percentiles that is normalized using 3 years of data in patients 6 years or older, see Corvol et al 2015 for details. For the lung function response to the CFTR modulator, we followed the exclusion criteria of Strug et al 2016: all participants included for modulator lung response analysis had a baseline measurement between 30 and 96 FEV1pp measured within 3 months prior to, or on, the treatment initiation date and were on commercial dose and we have a Supplementary Table 1 for the number of participants removed according to the exclusion criteria for this CFTR modulator study. QC for UKBiobank data followed the steps in Shrine et al, 2019 with minor changes.
Replication	This is not the first study to work on rs7512462 with lung function in CF, our findings in this paper expanded the previous reports that rs7512462 correlates with improved CFTR function. Here we replicated rs7512462 association with lung function across many different cohorts which was the major objective of the paper.
Randomization	This is not randomization study but rather an observational study. Previous work identified covariates important for inclusion in the models, and we further included them here in all analyses as well as adjusting for population structure where applicable.
Blinding	Blinding is not relevant to this study, as all patients with a cystic fibrosis (CF) diagnosis from participant clinic centers are eligible to participate in the study.

Reporting for specific materials, systems and methods

We require information from authors about some types of materials, experimental systems and methods used in many studies. Here, indicate whether each material, system or method listed is relevant to your study. If you are not sure if a list item applies to your research, read the appropriate section before selecting a response.

Materials & experimental systems

n/a	Involved in the study	
☒	☐	Antibodies
☒	☐	Eukaryotic cell lines
☒	☐	Palaeontology and archaeology
☒	☐	Animals and other organisms
☐	☒	Human research participants
☒	☐	Clinical data
☒	☐	Dual use research of concern

Methods

n/a	Involved in the study	
☒	☐	ChIP-seq
☒	☐	Flow cytometry
☒	☐	MRI-based neuroimaging

Human research participants

Policy information about studies involving human research participants

Population characteristics	As of today, the CGMS has enrolled 3,257 participants. These individuals have various causal CFTR genotypes that are reflective of the Canadian CF population with 50.3% as homozygous Phe508del, 53.6% male, mean age at 34.9 yrs (range: 3.99-90.8yrs).
Recruitment	The CGMS includes 35 clinics in 9 provinces across Canada. All the patients in these clinics diagnosed with CF are eligible for participating to the study. Written informed consent was obtained from all participants or parents/guardians/substitute decision makers prior to inclusion in the study.

Ethics oversight	The Hospital for Sick Children

Note that full information on the approval of the study protocol must also be provided in the manuscript.
